# Key challenges in providing assisted dying in Belgium: a qualitative analysis of health professionals’ experiences

**DOI:** 10.1177/26323524251318044

**Published:** 2025-02-06

**Authors:** Madeleine Archer, Lindy Willmott, Kenneth Chambaere, Luc Deliens, Ben P. White

**Affiliations:** Australian Centre for Health Law Research, Faculty of Business and Law, Queensland University of Technology, 2 George Street, Brisbane City, QLD 4000, Australia; Australian Centre for Health Law Research, Faculty of Business and Law, Queensland University of Technology, Brisbane City, QLD, Australia; End-of-Life Care Research Group, Ghent University and Vrije Universiteit Brussel, Ghent, Brussels, Belgium; Department of Public Health and Primary Care, Ghent University, Ghent, Belgium; End-of-Life Care Research Group, Ghent University and Vrije Universiteit Brussel, Ghent, Brussels, Belgium; Department of Public Health and Primary Care, Ghent University, Ghent, Belgium; Australian Centre for Health Law Research, Faculty of Business and Law, Queensland University of Technology, Brisbane City, QLD, Australia

**Keywords:** assisted dying, Belgium, euthanasia, qualitative research, regulation

## Abstract

**Background:**

Assisted dying or ‘euthanasia’ has been legal in Belgium since 2002. Extensive research has been conducted which investigates Belgian euthanasia practice, however, the current challenges that health professionals face when providing euthanasia are not well known. This knowledge is important for evaluating the current system, especially in light of recent developments in Belgian euthanasia law and practice including judicial decisions, legislative amendments and research highlighting the complexity of its governing regulatory framework.

**Objectives:**

This study investigates the key challenges that health professionals experience when providing euthanasia in Belgium.

**Design:**

A qualitative interview study with reflexive thematic analysis.

**Methods:**

Twenty interviews were conducted between September 2022 and March 2024 using Microsoft Teams videoconferencing. Eligible participants were physicians and nurses who spoke English or Dutch and who had been involved in the euthanasia assessment of at least two patients in the past year.

**Results:**

Four themes were generated: (1) the framing of the euthanasia legislation poses challenges; (2) providing euthanasia can place considerable burdens on health professionals; (3) clashing views about euthanasia can hamper opportunities for balanced discussions and (4) euthanasia and processes relating to euthanasia are not always well-understood.

**Conclusion:**

This study highlights the numerous and varied challenges physicians and nurses experience when providing euthanasia in Belgium, even 20 years after its law passing. This study contributes to a wider understanding of universal challenges associated with providing assisted dying and sheds light on issues specific to Belgium. The results provide an opportunity for policymakers to take action to better support providers to manage these challenges, including through a formal review of the legislation and the broader system.

## Plain language summary

What aspects of providing euthanasia in Belgium do health professionals find challenging?

This study investigates the challenges that doctors and nurses experience when they are involved in providing euthanasia in Belgium. Euthanasia is when a person makes a voluntary request to a health practitioner for help to die. Belgium has permitted euthanasia since 2002. Under the law, doctors assess whether patients are eligible to access euthanasia. Nurses assist doctors and have other important roles in caring for patients who access euthanasia. Much research explores Belgian euthanasia practice. However, research has not considered the many challenges doctors and nurses experience when assisting patients to access euthanasia. These findings are an important source of knowledge when euthanasia law and practice is reviewed. Measures can be implemented to address these challenges, improving patients’ and providers’ experiences of the euthanasia system. We conducted interviews with 20 doctors and nurses. They described four main challenges: the framing of the euthanasia legislation poses challenges; providing euthanasia can place considerable burdens on health professionals; clashing views about euthanasia can hamper opportunities for balanced discussions; and euthanasia and processes relating to euthanasia are not always well understood. Doctors and nurses should be supported to manage these challenges. Measures should also be implemented to manage these challenges, including evaluating and, if needed, making changes to the law; implementing mandatory training for doctors and nurses involved in providing euthanasia; and providing community education on euthanasia and the legal process. These findings are also important for other places which are considering legalising euthanasia in the future.

## Introduction

Assisted dying (‘AD’) is becoming increasingly legalised internationally.^1^ There are now over 20 jurisdictions that permit AD in some form.^2,3^ Belgium has one of the longest-standing AD legal frameworks internationally. The *Act on Euthanasia* (‘the Act’) was passed in 2002, so for the last two decades doctors have been permitted to assist patients to die when the legal requirements are met (Table 1). A significant body of empirical research has investigated Belgian AD practice and regulation; few other AD regulatory systems are as well established and researched.

**Table 1.** Legal process for access to euthanasia under the Belgian *Act on Euthanasia*.

Basis for request	Patient request	The attending physician	The (first) independent physician		The (second) independent physician/practitioner	Additional requirements
Contemporaneous request	A written request in the prescribed form	• Assesses whether the: • Patient is legally competent, a legally competent emancipated minor, or a minor with the ‘capacity for discernment’ and conscious at the moment of making the request • Request is voluntary, well-considered and not the result of external pressure • Patient is in a medically futile condition of constant and unbearable physical or mental suffering that cannot be alleviated, resulting from a serious and incurable condition caused by illness or accident (limited to physical suffering and death will result in the foreseeable future for minors with the capacity for discernment) • Informs the patient about their health condition and life expectancy, their request, existing therapeutic and palliative options and their consequences and has several conversations with the patient spread over a reasonable period of time • Consults the patient’s nursing team, if relevant and discuss the patient’s request with their relatives if the patient chooses to do so • Refers the patient for at least one further consultation	Reviews the patient’s medical record, examines the patient and assesses the patient’s suffering and the non-alleviable nature of their condition. They provide a report to the attending physician on their findings	**Patient is expected to die in the foreseeable future**	–	–
				**Patient is not expected to die in the foreseeable future**	Is a psychiatrist or a specialist in the patient’s condition. They review the patient’s medical record, examine the patient, and assess the patient’s suffering that cannot be alleviated, and the voluntary, well-considered and repeated nature of the request. They provide a report to the attending physician	A 1-month waiting period must be observed between the request and the performance of euthanasia
				**Patient is a non-emancipated minor**	Must be a child and adolescent psychiatrist or psychologist and they must review the patient’s medical record, examine the patient, ascertain their capacity for discernment and certify this in writing	The attending physician informs the patient and their legal representatives about the results of the consultation, further information and obtains the patient’s and legal representatives’ consent with respect to the request
Advance request	Advance request for euthanasia in the prescribed form	• Assesses whether the: • Patient suffers from a serious and incurable condition, caused by illness or accident • Patient is no longer conscious • Patient’s condition is irreversible given the current state of medical science • Consults the patient’s nursing team and discusses the contents of the advance request with them • Discusses the patient’s request with any confidant designated by the person in their advance request • Refers to the patient for further consultation and informs the confidant of the results of this consultation	Reviews the patient’s medical record and examines the patient. They provide a report to the attending physician on their findings	-	-	-

Evidence suggests the Belgian system is working well in several respects. Official data from the national oversight body, the Federal Control and Evaluation Commission on Euthanasia (‘FCECE’) documents just one referral to the public prosecutor since 2002 and does not raise concerns of abuse.^4^ Research demonstrates that health professionals generally view the system positively.^5–7^ However, there is also evidence highlighting some complexities within the system. Some, albeit limited, evidence indicates that there is some non-compliance with the legal framework. This usually involves failures to report performed cases of AD to the FCECE,^7–9^ to obtain a patient’s request for AD in writing and to obtain the advice of a second, independent physician on the patient’s eligibility.^10–12^ There is also some evidence of nurses administering the life-ending medication though under the law they are not permitted to do so.^13^

Recent research illustrates the complex regulatory framework governing AD in Belgium and describes the implications of this complexity for patients’ and providers’ experiences of AD.^14–16^ There have also been developments in Belgian AD or ‘euthanasia’ (as it is known there) law and practice in recent years. Significant amendments to the law were made in 2014, 2020 and 2024. In 2020, Belgium’s first criminal trial in relation to euthanasia was decided. This case was escalated to the constitutional court in 2022. Another case was heard in the European Court of Human Rights in 2022. These cases have not yet resulted in any criminal or civil sanctions for health professionals. However, they received significant attention in the media and exposed problems with the legislative control and oversight mechanisms.^17–20^

Despite the volume of research investigating euthanasia in Belgium, the broad spectrum of challenges that health professionals experience when providing euthanasia has not been systematically studied. Some challenges have previously been identified, such as difficulties physicians experience applying the Act for patients whose request is based on mental disorder^5,21,22^ and the emotional experiences of nurses when involved in providing euthanasia.^6,23,24^ A more comprehensive investigation of these challenges offers the opportunity to evaluate current law and practice and advance an understanding of providers’ experiences of the system. Recent developments in Belgian law and practice provide a useful juncture to undertake this investigation. This study addresses the research question: what are the key challenges that health professionals experience when providing euthanasia in Belgium?

## Methodology

This study is part of a larger international study examining AD regulation in Australia, Canada and Belgium.^25^ We adopted a critical realist position in this research.^26^ Semi-structured interview research design was used to obtain insights from health professionals in Flanders and Brussels.

### Participant eligibility and recruitment approaches

Health professionals were eligible to participate if they were a physician or nurse who spoke English or Dutch, and who had been involved in the euthanasia assessment of at least two patients in the past year. This latter requirement was to ensure that participants’ experiences providing euthanasia were recent. Only physicians can assess patients’ eligibility and administer the life-ending medication. Nurses were also recruited as they can be involved in euthanasia decision-making and have an important caregiving role.^13,23,24^

We recruited participants directly through the professional networks of the Belgian authors (KC and LD), advertisements disseminated by relevant organisations and using a snowball approach. We purposively selected participants to capture heterogeneity in participants’ experience providing euthanasia, gender identity, location of practice, profession, and cultural and ethnic backgrounds. Participant consent was obtained prior to participation.

### Data generation

Interviews were conducted between September 2022 and March 2024. We interviewed participants at a time and location of their choosing using Microsoft Teams videoconferencing. MA led the interviews conducted in English (with LW co-interviewing in several interviews). Participants could also indicate that they would prefer to participate in Dutch or be supported to participate in English (supported English). MA led the interviews in supported English, assisted by KC or LD (Dutch-speaking members of the research team). KC led the interviews undertaken in Dutch. Participant quotations are included in the results, below. In some cases, they have been edited to clarify the participant’s intended meaning in English and to preserve participant anonymity. Their meaning has not been altered.

We developed and used an interview guide in which the prompts were tailored for each participant (including their health profession and work setting; Supplemental File 1). The interview guide was structured to obtain participants’ insights on each part of the euthanasia process (including their experience of challenges) from its beginning to end (from identifying a patient’s request through to administration and aftercare). We also asked participants to consider the euthanasia system in a general sense and identify both the best and more challenging parts of the system. The researcher leading the interview made field notes during and after the interview.

Interviews were recorded and transcribed verbatim by a professional transcription company. MA checked the accuracy of the transcripts against the recording and returned the transcripts to participants, giving them the opportunity to add further or remove reflections.^27^ The Dutch interviews were then translated into English by a translator with experience researching euthanasia.^28^

Interviews ceased when ongoing analysis demonstrated that sufficient ‘information power’ had been reached.^29^ This decision was made after discussion within the research team and was based on the study’s broadly framed research question, inclusion of participants with both considerable and limited experience providing euthanasia, and preliminary analysis of the data.

### Data analysis

The transcripts were thematically analysed by MA using Braun and Clarke’s reflexive approach to thematic analysis facilitated by NVivo software (QSR International, release 1.6.1).^30^ This approach reflected the study’s exploratory research question and facilitated an inductive orientation to generating semantic and latent themes.^30–32^ MA read the transcripts in full and made notes without coding to ensure data familiarisation. MA then coded the transcripts. The first 10 transcripts were coded twice to generate a coding framework that was applied to facilitate analysis of the remaining transcripts, which were also coded twice. MA then generated initial themes by sorting and integrating codes into patterns of shared meaning.^33^ The research team discussed the initial themes at several stages, which resulted in some themes being merged, adapted and ultimately finalised. In particular, discussions about the use of illustrative participant quotations were used to inform theme development.

Reflexive notes were made by MA after each interview, notes were made during data familiarisation, and field notes were used to inform the coding and theme generation processes.

### Reporting of the study

This study is reported in accordance with the consolidated criteria for reporting qualitative studies (‘COREQ’; Supplemental File 2 contains a completed COREQ checklist).^34^ Supplemental File 3 reproduces recommendations made in the Reflexive Thematic Analysis Reporting Guidelines^35^ with a corresponding discussion of how each recommendation has been reflected in the conduct and reporting of this study (where possible and appropriate).

## Results

### Participant characteristics

We conducted 20 interviews. The median interview length was 94 min (range: 65–111 min). Participants’ demographic information is reported in Table 2. While we attempted to recruit individuals with a range of cultural and ethnic backgrounds, all participants identified that they were of Belgian Dutch-speaking ethnicity. Most participants were comfortable speaking in English in the interview (*n* = 14), with some participating in Dutch (*n* = 2) or supported to participate in English (*n* = 4).

**Table 2.** Participant demographic information.

Participant characteristic	No. participants (*N* = 20)
Gender identity	
Female	11
Male	9
Age category (years)	
<30	1
31–40	2
41–50	7
51–60	5
61–70	2
>70	3
Health profession	
Physician – general practice specialty	5
Physician – medical specialist	9
Nurse	6
Location of primary practice	
City	11
Town	7
Rural municipality	2
Region of practice	
Flanders	19
Brussels	1
Main setting of practice	
Community	7
Hospital	11*
End-of-life consultation centre	2
Years of experience	
1–10	4^$^
11–20	4
21–30	4
31–40	4
41–50	4
Number of euthanasia cases involved in providing^‡^	
<10	1
10–19	2
20–29	2
30–49	3
50–99	4
100–500	3
>500	5

* One participant who currently works in the hospital setting also has significant (though previous) experience working in the community setting.

$ One participant indicated that they had worked in their current role for 9 years, and it was not clear if their work history extended past this role. The participant could not be contacted to clarify.

‡ Nurse participants had been involved in providing euthanasia in 30–49 cases (one participant), 50–99 cases (three participants), 100–500 cases (one participant) and >500 cases (one participant).

### Themes on key challenges in providing euthanasia

**Figure 1.**
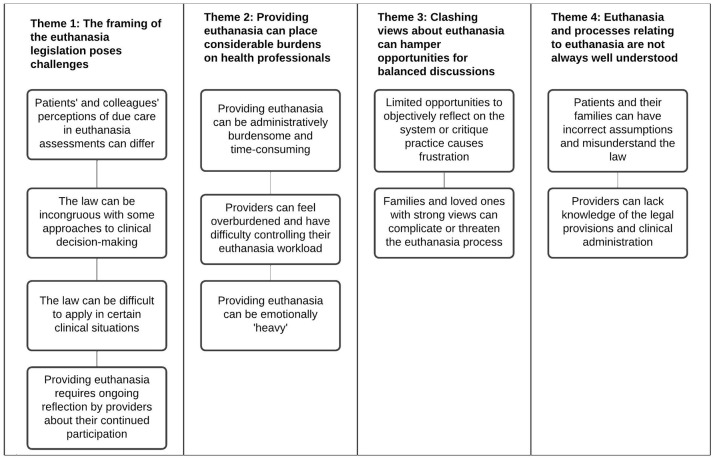
List of themes and subthemes.

We generated four themes in the analysis, each with subthemes, reflecting the key challenges participants reported when involved in providing euthanasia. We use ‘involved in providing’ in a broad sense to describe all the actions that a health professional might engage in including having discussions about euthanasia, caring for a patient who has made a request and administering the life-ending medication (referred to below as ‘administration’). Figure 1 provides a list of the themes and subthemes.

While the focus of this article is on the challenges participants identified with providing euthanasia, it is important to acknowledge that all participants in this study reported that they appreciate that euthanasia is a legal possibility in Belgium. All reported being generally satisfied with the Belgian euthanasia system and viewing it positively, notwithstanding their experience of the challenges reported.

## Theme 1: The framing of the euthanasia legislation poses challenges

All participants identified challenges with the framing of the euthanasia legislation and its eligibility criteria. They reflected that the framing of the legislative provisions gives scope for providers to customise their approach to providing euthanasia. In addition, the broadly framed eligibility criteria provide scope for a wide range of patients to access euthanasia. The resulting challenges are reflected in four subthemes.

### Subtheme 1a: Patients’ and colleagues’ perceptions of due care in euthanasia assessments can differ

Numerous physician participants reported that they employ measures which go beyond the various legal requirements to exercise due care and clinical caution in the euthanasia assessment process. Most participants working in hospital palliative care support teams (‘PCSTs’) described that their hospital applies the ‘palliative filter’ in the assessment process. This refers to the PCST being involved to some extent in all requests for euthanasia; their role can differ depending on the institution but includes scrutinising whether the legal requirements have been followed and ensuring that the patient has had the opportunity to consider their remaining palliative care and therapeutic options. Many participants reported that patients or others in the community can view these measures as an attempt to delay or thwart their access to euthanasia.

Because here, and I think that’s the problem in Belgium, that it’s very polarised. . . And if you start asking questions, they *[patients and their family members]* immediately say, ‘OK, you’re Catholic, so you don’t do it. . . this is your way to just stop things’. And then it’s very difficult to have a dialogue. Because that’s really not what we are trying to do. – Participant 13, medical specialist

I know there’s a lot of fuss about: ‘you’re stricter than the law, that’s not right, that’s not allowed’ *[by applying the palliative filter]*. . . We follow the law, but we also take care of the physicians who have to do it. Because at the end of the day. . .in our *[institution]* there’s a small group, maybe 10, 15 doctors who will effectively perform euthanasia. . . It’s not just the doctors who carry the load of that, but also the nurses in the department aren’t used to allowing euthanasia to simply go ahead *[without exercising due care]*. . .. – Participant 9, nurse

Some participants described employing other measures or considerations which patients can view as obstructive, rather than recognising that health professionals were aiming to provide a careful or quality euthanasia trajectory. This includes providers taking time in the process to develop a relationship with the patient before progressing further or educating patients that euthanasia may not be the most suitable option (clinically) for them.

I always talk about the possibility that euthanasia can be impossible or can be not the best choice because disease is developing in a direction where euthanasia is really not the best solution. And sometimes patients react or families react, ‘Oh, you’re negative about euthanasia?’ ‘No, no, no, I’m not negative about euthanasia, but it’s not, in every case, the best solution’. – Participant 4, general practitioner

Several participants reported that their colleagues can also react negatively to their attempts to exercise clinical caution. Several described their role as the independent (second) physician being viewed by the attending (first) physician as a formal requirement rather than a valuable check on decision-making.

The *[first]* doctor has promised, has made an agreement with the patient and his proxies *[that he will provide euthanasia]* but there has to be a second physician. I’m not involved in the process *[as the second physician]*, I am a passenger sometimes, I think. The *[first]* doctor has already in his mind it will be performed. And the second advice is something that the law requires, but that has no value. – Participant 6, general practitioner

Some participants reported having negative views of some colleagues’ approaches to conducting euthanasia assessments and consider that they do not always exercise due care. Several participants reported that they consider providers who facilitate ‘urgent’ euthanasia requests to lack clinical caution, believing there is no opportunity in such cases for all parties to consider the request to the necessary extent. One participant referred to the different methods that physicians use for the administration of life-ending medication and that their favoured method of an intravenous infusion is more clinically sound than other approaches used by their colleagues. However, this participant expressly identified that such variation is inherent in all medical practice, and not unique to euthanasia provision.

### Subtheme 1b: The law can be incongruous with some approaches to clinical decision-making

Many participants expressed that the Act and its legal processes can be incongruous with their usual approaches to clinical decision-making. Many identified that the legal process, characterised by separate, independent consultations by physicians, sits awkwardly with the multidisciplinary shared decision-making processes that normally characterise their clinical decision-making, especially prevalent in the institutional and palliative care contexts.

. . . physicians in Belgium find this extremely annoying that an independent assessment has to be made. Because usually, we all work in teams. We are not solo practices. So we work in teams so there are other physicians in the team, nurses, and psychologists. So we decide in the team whether or not to do this and then having to ask someone completely outside the team to reassess the patient, it feels unnecessary. . .. – Participant 3, medical specialist

Many participants lamented the limited role afforded to nurses in the Act, which one nurse participant described as ‘literally zero’, despite the often significant and varied roles nurses can have in the euthanasia process. Given the absence in the Act of clear prescriptions around nurses’ proper roles in the euthanasia process, one nurse participant identified that sometimes nurses need to have a ‘pit bull attitude’ to be involved in a given case, which should not be required.

As discussed above, many participants identified that euthanasia provision in urgent cases is not congruent with their views of best-practice euthanasia decision-making and the role of euthanasia as an end-of-life option. Other participants considered that the speed with which euthanasia can be performed in Belgium is an asset of the system, and entirely consistent with what they consider to be the aim of the legislation. This is because euthanasia can be provided very quickly for patients who are enduring intolerable pain and suffering, so their suffering does not need to continue longer than necessary.

Almost all participants discussed the legislative provision which provides that a patient’s family members do not have to be involved in the patient’s euthanasia assessment process. Many participants identified that this provision is inconsistent with best-practice end-of-life decision-making, specifically relating to euthanasia, which is to involve the family in the usual course. One participant identified that some patients are aware of this legislative provision, and ‘use it against’ the treating team to authorise their choice not to tell their family they are accessing euthanasia. While this participant experiences frustration when this happens, they, and all other participants acknowledged that the legislation could not be framed differently to overcome this, outlining limited but legitimate circumstances in which the family should not be involved.

### Subtheme 1c: The law can be difficult to apply in certain clinical situations

Many participants reported that some of the legal provisions are challenging to apply in specific clinical situations. First, many participants identified that it is hard to assess patients whose request is based on mental disorder, specifically whether all treatment options have been exhausted, and whether their condition is incurable. Almost all participants referred to these cases as ‘complex’. One participant identified that ‘it is never clear that you cannot help them anymore’. This participant described that the overburdening of end-of-life consultation centres such as Ulteam and Vonkel reflects the difficulty that providers have assessing these patients; their caseloads primarily consist of patients with mental disorders and in the early stages of dementia.

Second, under the Act, patients who are not expected to die in the foreseeable future undergo a different assessment trajectory than those who are. Most participants identified that it is difficult to assess whether the patient is or is not expected to die in the foreseeable future, as ‘foreseeable future’ is subjective.

I think that we already have very clean legislation. . . *[but]* . . . there has to be some more clarification, surrounding. . . what is terminal and non-terminal. – Participant 17, nurse, translated

Finally, several participants reported difficulty understanding the legislative circumstances in which an advance request for euthanasia can operate. Some were unsure whether the request could apply when the patient is suffering from advanced dementia (and lacks capacity) or is in the ‘dying phase’ of their illness. Uncertain whether to follow the advance request or not, participants reported either not following it, as they considered that to be safest, or relying on their ethical judgement at the time to make this decision. Almost all participants identified that the Act is unclear in this respect, and some noted that attempts by advisory bodies to clarify the issue have not provided a conclusive answer.

### Subtheme 1d: Providing euthanasia requires ongoing reflection by providers about their continued participation

Many physician participants expressed that their degree of comfort providing euthanasia is not fixed and that they reassess it for each patient, almost on a case-by-case basis. The clinical characteristics of patients who may be eligible under the Act differ so much that a participant may be comfortable to assist one patient, but not the next. This is due to the broad eligibility criteria in the Act.

Some participants reported needing to undertake an ongoing ‘ethical questioning’ to determine whether they are comfortable being involved at all for the specific patient, and if so, which role they feel comfortable taking. For example, some participants described being comfortable acting as the independent but not attending physician, as the scope of this role is more confined including that the independent consultant is not expected to administer the life-ending medication.

I think that with every patient that gets euthanasia, I need to be comfortable with performing it. And with a lot of patients who are not terminally ill, I have more of an ethical difficulty. So up to this point, I have not been comfortable with performing euthanasia in those cases. So I have referred all those non-terminal patients to one of my colleagues. But I have been second physician in some of those cases because then I don’t have to do it. . . and that’s fine for me. But I don’t want to be the one performing euthanasia in the non-terminal cases. – Participant 11, medical specialist

## Theme 2: Providing euthanasia can place considerable burdens on health professionals

This theme, comprised of three subthemes, describes participants’ perceptions that providing euthanasia can be practically and emotionally burdensome.

### Subtheme 2a: Providing euthanasia can be administratively burdensome and time-consuming

Physicians must complete and submit a registration form to the FCECE after the patient has died. Some physician participants, generally those who were less experienced in providing euthanasia, reported that this process is administratively burdensome, complicated and time-consuming. One nurse participant reported observing a physician who had provided euthanasia for the first time taking over 2 h to complete it. Other, more experienced, physician participants reported that this aspect is one of the most straightforward parts of providing euthanasia.

Almost all participants reported that providing euthanasia is time-consuming. The assessment process involves conducting numerous, often lengthy consultations with the patient. This work is often undertaken outside regular working hours. For those working in the community setting, it can involve travelling some distance to see the patient.

### Subtheme 2b: Providers can feel overburdened and have difficulty controlling their euthanasia workload

Several participants described feeling overburdened or unable to effectively control their euthanasia workload. Several participants reported being asked to step into a euthanasia assessment process to replace an unavailable colleague, often urgently, as the eligible patient’s condition had rapidly deteriorated, and they wanted to have the life-ending medication administered. Some general practitioners reported that they have become known as the practitioners in the practice or local region who is comfortable providing euthanasia, hence they receive a significant volume of referrals to assess their colleagues’ patients.

Many participants perceived a reduction in the pool of euthanasia providers following a criminal trial of three physicians in relation to euthanasia.^36^ They expressed that this has contributed to the overburdening of the remaining euthanasia workforce, particularly in the community setting. Several general practitioner participants reported that they are aware they can refuse to be involved, but they have a strong sense of professional responsibility which means they have difficulty refusing. They cited concerns about the consequences of refusing to assess a patient: the patient having difficulty finding another physician willing to provide, worries the patient may attempt to commit suicide, losing continuity of care so close to the end of life and delays exacerbating already acute suffering.

. . . we are in this kind of funnel in my region where everybody is just knocking on our door. And we tell them, ‘No, we cannot take new patients, nobody’. And then sometimes these cases trickle through, I don’t have the guts to tell our terminally ill patients, ‘No, you look further *[for another practitioner to facilitate their request for euthanasia]*’. Because I know they will not find anyone, that’s the problem. – Participant 2, general practitioner

One participant reported that there is a dearth of psychiatrists willing to assess patients for euthanasia. This means for psychiatrists the issue is even more pervasive, and they face considerable pressure to continue assessing patients.

### Subtheme 2c: Providing euthanasia can be emotionally ‘heavy’

Most participants described providing euthanasia as emotionally ‘heavy’. They identified that it is particularly so when receiving a patient’s request for euthanasia and during administration. These emotional challenges appeared to be most acute for less experienced providers, those who were infrequently involved in providing euthanasia, and those who did not work in team-based environments. Cases in which the patient’s request is motivated by a mental disorder were also reported as being particularly emotionally challenging. Some very experienced physician providers did not describe the emotional challenges associated with providing euthanasia. Participant quotes illustrating this subtheme are presented in Table 3.

**Table 3.** Participant quotes describing how providing euthanasia can be emotionally burdensome in certain situations.

Relevant aspects of the euthanasia assessment process	Illustrative quote(s)
Receiving a euthanasia request	‘*And then, on day one, you get two patients with their paper, like, “OK doctor, I want euthanasia.” And then I, my first, my feeling is like, swallow. OK. Calm. Just sit down. And then you* [say to yourself] *“OK, relax.” And then I explore, and I think . . .*’. – Participant 13, medical specialist
	‘*And there are also colleagues who are like, “OK, the concept of euthanasia is OK for me, but please don’t ask me the question personally because I’m panicking, and I don’t know what to do*”’. – Participant 11, medical specialist
	‘*I hope I don’t have to do it, of course. So it’s not a topic I offer so easily, but normally if we are in the setting of palliative care, I do it* [raise euthanasia]. . . *it’s a process and it’s tough. So if I can avoid it, yes, please. But of course if it’s needed, I will do it. I do also other things I don’t like, but I mean, it’s not something I want to do normally*’. – Participant 10, general practitioner
Referring a euthanasia request to the palliative care support team	‘*Usually there’s a kind of a question from someone who wants to die, and there’s a kind of panic, and then they already phone us before even the treating physician knows about the question. Or sometimes the treating physician knows, but he says, like, “OK, I don’t do euthanasia,” or, “This is for the Palliative Care Team”*’. – Participant 13, medical specialist
	‘*In our* [institution]*. . . nurses are still really stressed about it. If a patient says something about life ending or euthanasia, it’s a lot of stress. And then they call us and then they say, “We have a patient here who has asked about euthanasia or has asked about other documents about life ending. Can you please come? Because we don’t know how we have to do it*”’. – Participant 15, nurse
Administration of the life-ending medication	‘*. . .performing euthanasia is always difficult, it always gives some kind of storm inside of you*’. – Participant 14, medical specialist
	‘*Every time again, I’ve experienced so many now, every time again it goes to my core. Incredibly deep*’. – Participant 9, nurse (translated)
	‘*. . .for us as doctors, I mean, I’ve done euthanasia, but I remember every single patient. It’s a bit of a – yeah, it hurts my soul every time I do it. It sounds a bit dramatic, but it really does*’. – Participant 11, medical specialist
Encountering patients who consider that euthanasia is easy for providers to administer	‘*I think to perform euthanasia with a patient, it’s something very specific you do. It’s not like another injection, for instance. Technically it is. But on the human level it isn’t. So I think it’s important to have the, yeah, to have some relationship with the patient. And in a way that you’re at the same level, that the patient also recognises the fact that for you as a doctor it’s not just a technical thing. And if, yeah, for me, if a patient tries to reduce me to a technician who has to fill out his individual requests in any way, yeah, that’s for me, a red light, I think*’. – Participant 4, general practitioner

Several participants described that receiving a patient’s request for euthanasia is a significant, often emotional moment. Participants have observed providers’ reactions to receiving a request ranging from surprise or discomfort to feeling overwhelmed, fear, stress or panic. Participants working in PCSTs reported that a very common response for treating teams is to engage in immediate support-seeking and make a referral to the PCST. In some cases, this is made before even the treating physician is informed.

The administration of the life-ending medication (or assisting its administration) was reported by participants as ‘a burden’ and ‘very emotional’ owing to the significance and finality of the act. For this reason, most participants reported that they have a policy of ensuring that euthanasia is not administered by a physician on their own. Participants described that maintaining professionalism and composure during administration can be difficult and that emotional reactions must be reserved for a private setting after the patient has died. The administration was reported as being significantly more emotional when there were technical challenges with the administration, such as problems inserting the cannula.

Many participants reported having heard patients refer to euthanasia as the ‘best’, ‘easiest’ or ‘simplest’ way of dying. Each expressly identified that they reject this reductionist view of their role in administration, given its significance.

## Theme 3: Clashing views about euthanasia can hamper opportunities for balanced discussions

This theme, comprising two subthemes, describes participants’ reflections about the polarising nature of euthanasia, both in terms of the general societal debate and within specific clinical cases. Participants identified that this can hinder their ability to have productive and nuanced discussions about euthanasia in public and private spheres.

### Subtheme 3a: Limited opportunities to objectively reflect on the system or critique practice causes frustration

Almost all participants reported being frustrated that there is no value-neutral forum in which they might critically discuss euthanasia law and practice. Participants expressed that critically reflecting on, evaluating or suggesting improvements to the system would be perceived as expressing an ideological opposition to euthanasia. Participants expressed this hostility reflects the political landscape surrounding euthanasia in Belgium. Participants also reported fearing that suggesting legislative reform might lead to an unpredictable political debate that could result in adverse consequences, such as repealing the law.

Changing the law means a lot of problems because when you start changing the law, other parties will try to make other changes too and then it’s what we call Pandora’s Box. Maybe you end up with no legislation anymore. – Participant 5, general practitioner

Participants expressed mixed views concerning possible expansion to the Act’s scope. Some were supportive of including a wider range of patients under the Act, but they did not know if they would be comfortable to provide euthanasia for those patients, nor how the legislative framework could appropriately reflect the change. Most participants strongly called for a formal, objective review of the law to be undertaken, partly to review current law and practice, and partly as a first step for considering expansion to the Act’s scope. Many identified that this review would present an opportunity to provide an objective evidence-base that is not coloured by the biases that permeate public discussions.

The law is not sufficient in protecting people. And I think before we enlarge, because there is now a discussion going on about should people with *[advanced]* dementia, for instance, have access under certain conditions, to euthanasia. I think before we enlarge the groups, I think we should have a proper discussion about the problems nowadays. And before that evaluation, I think it’s not a good idea to enlarge the groups. – Participant 4, general practitioner

### Subtheme 3b: Families and loved ones with strong views can complicate or threaten the euthanasia process

Patients’ families and loved ones can have strong views in relation to the patient’s choice to access euthanasia. Many participants reported that these views can complicate the process and cause providers to feel threatened regarding their involvement.

Some participants reported that where families support the patient’s decision to access euthanasia, the strength of this conviction can complicate the euthanasia process. Families can view the required assessment process as obstructive and unnecessary, or an illegitimate attempt to preclude access. One physician participant reported a situation in which a patient died before they could obtain the independent advice, following which the patient’s family blamed the physician and their assumed obstructiveness for the patient having been unable to access euthanasia. In this way, this theme intersects with theme 1a, described above, which describes patients’ views of ‘due care’ conflicting with those of physicians.

Many participants reported fearing ‘backlash’ when family members not appraised of the patient’s decision to access euthanasia are informed that euthanasia has been administered. Some participants have experienced families accusing them of improperly performing euthanasia, threatening legal action or involving the press in these situations. One participant described having provided euthanasia for a patient where the participant (unintentionally) did not include the patient’s grandchild in the discussion. The patient and their grandchild had previously enjoyed a very close relationship. Having been informed that their grandparent had died, the grandchild was angry and blamed the treating team for having ‘killed’ their grandparent.

Some participants also reported that families can be very respectful and supportive of the patient’s decision, even if they do not agree with it.

## Theme 4: Euthanasia and processes relating to euthanasia are not always well-understood

This theme, comprising two subthemes, describes participants’ reflections that patients, families and health professionals do not always understand the euthanasia legislative framework which can cause complications and frustration. Participant quotes illustrating both subthemes are presented in Table 4.

**Table 4.** Participant quotes describing patients’ and practitioners’ lack of knowledge about euthanasia and the legal process.

Subtheme	Illustrative quote(s)
Patients’ lack knowledge and can misunderstand euthanasia and the legal process (Subtheme 4a)	‘*We often have patients who have these papers filled in, and who think that, that this paper will give them the right to get euthanasia. So, they say: “All my papers are in order, so it’s all clear.” But that’s sometimes confusing for patients because, for this paper, they have to be in coma. So, it’s not like, if they are still able to talk to us, it’s not like, yeah, they shouldn’t talk to us anymore. So, so we, so that, that’s one of the first misunderstandings we often have to solve with our patients*’. – Participant 19, medical specialist
	Describing the option patients have of completing a document in which they make an advance request for euthanasia to which a physician may only adhere if the patient enters a state where they become permanently unconscious: ‘*. . .it gives a lot of stress and confusion and conflict situations in settings where you have other stuff to do. It’s time to say goodbye, it’s time to perform good palliative care in all aspects of it. And you’re fighting over papers* [which] *have no value. So that needs, I think those papers are the worst part of the law, actually. Yeah, because people really think that they have their business taken care of, how they’ve been to the, their will, their last will in order, they have been to the undertaker for their funeral, they have performed everything. And they have had their papers in order. And then the papers are worth nothing*’. – Participant 11, medical specialist
	*Setting up an advance directive, that’s not straightforward. . . . We hear unusual things sometimes. Really, the training* [of physicians] *has to improve on that. . .. knowledge of the surrounding legislation really is lacking. We hear a lot of doctors say the strangest things to their patients*. – Participant 17, nurse (translated)
Practitioners’ lack of knowledge about the law and their obligations (Subtheme 4b)	‘*I have now had two negative judgments from a specialist that just are incomprehensible, they just make no sense. But that is really due to ignorance, you know, that they give those judgments. That’s a real shame. I think there’s a lot of work to be done in that area when it comes to the training of doctors. . . The negative judgment, the two negative judgments were from a psychiatrist who claimed Parkinson’s wasn’t an incurable condition. I’ve never seen a single Parkinson’s patient recover. . .*’. – Participant 17, nurse (translated)
	‘*. . .for us that’s the, daily is a little over the top, but weekly frustration still that they* [physicians] *are insufficiently educated in that and don’t know how the legislation works. And still doctors who, for example, think that an advance directive is enough, that’s pretty far from where we need to be, you know?*’ – Participant 17, nurse (translated)
	‘*We have a few doctors here in the hospital who if you mention euthanasia – you lost them. And sometimes they refer to another doctor, but sometimes they don't, until we said – you are required to refer to another doctor. So now it’s changing, but it’s still a long process. And now they refer to other doctors, so they learn it*’. – Participant 15, nurse
Experience of technical difficulties with clinical administration (Subtheme 4b)	‘*I was so scared, I don’t do it, and I was so worried to get it outside the veins, and then it was not working immediately or whatever. So I was like, I’m terrified also about the practical thing*’. – Participant 10, general practitioner
	‘*I once had a nurse who was assisting the doctor and I was there because the family was very anxious and I was sitting with them. And the doctor put in the syringe and he tried to give the medication and it didn’t work. . . he looked like that* [gestures panicked expression]. *I just went to him, did something, I saw the problem*’. – Participant 12, nurse

### Subtheme 4a: Patients and their families can have incorrect assumptions and misunderstand the law

Almost all participants described that it takes time to educate patients and families to correct misunderstandings and fill knowledge gaps in relation to euthanasia. Some reported that patients and families commonly do not understand how palliative care and euthanasia differ. Most participants reported that patients believe drafting an advance request entitles them to access euthanasia at any time (and not only in the limited circumstances prescribed by the law, described in Table 1). Patients and their families can find it distressing to learn that access is very limited in cases of advance requests, and that euthanasia cannot be accessed if the patient has lost capacity as a result of having advanced dementia. In addition, most participants reported that patients often believe they are entitled to access euthanasia and do not know that a formal assessment process must be followed. One participant reported a patient becoming hostile when they asked them: ‘why do you want euthanasia?’

By contrast, some participants identified that certain patients appear to know the law very well. However, even where this is the case, participants reported needing to enhance the patient’s understanding of important contextual or clinical information.

Under the law you don’t have to involve the family. And patients they know it.. . . And they say to us, ‘Yes, but we don’t have to involve the family’. We say, ‘Yes, that’s the law. But we find that if you see your mother every day, we can’t help you to die without speaking with your mother. It’s impossible’. So it’s because of humane reasons that we involve the family. Or another reason is that sometimes it helps us to understand the patient. . .’. – Participant 20, medical specialist

### Subtheme 4b: Providers can lack knowledge of the legal provisions and clinical administration

Many participants reported experiencing frustration in situations in which their colleagues demonstrate a lack of understanding of the euthanasia law and legal process. Specifically, participants have observed their colleagues providing inaccurate information to patients about how long accessing euthanasia may take; believing that a patient’s advance request entitles them to access euthanasia now though are conscious and have capacity; and displaying a lack of knowledge of their legal obligations. One participant reported explaining to colleagues that they have a legal obligation to refer the patient where they have a conscientious objection to providing euthanasia. A nurse participant reported observing physicians incorrectly applying the eligibility requirements resulting in some eligible patients being considered ineligible (an illustrative quotation is contained in Table 4).

Some participants reported having difficulty with the clinical administration of euthanasia or being called to assist colleagues having difficulty. One general practitioner described a ‘horror scenario’ in which a suitable vein could not be found. The cannula was inserted and removed several times, with the patient and their family in visible distress. Most experienced participants, especially nurses, reported having a substantial role supporting less experienced physicians during administration (including preparing the medications and inserting the cannula). Though rarer, participants described that physicians and nurse colleagues may be called in to support in urgent situations, for example, when the IV line is clogged or an entrance cannot be found. Most nurse participants identified that these situations would have been prevented if the relevant, skilled personnel (nurses experienced in providing euthanasia) had been present. Nearly all participants expressed that training on euthanasia should be mandatory for all providers, with one participant suggesting that ‘it is a problem that everyone can perform’.

## Discussion

This study investigated the key challenges that health professionals in Belgium experience when providing euthanasia. Although generally positive about the Belgian euthanasia system, participating health professionals in this study identified several challenges they experience when providing euthanasia. Challenges are experienced by both more and less experienced practitioners working across all care settings.

This study adds to the Belgian and international AD literature. It identifies a broader spectrum of challenges than previously considered in Belgian studies.^6,23,24,37–41^ At one level, the challenges identified in this study reflect international experiences associated with providing AD. Evidence from other jurisdictions supports that assessing practitioners can find the AD legislation difficult to apply (consistent with theme 1c).^42–45^ Evidence of administrative burdens (theme 2a), the emotional toll associated with providing AD (theme 2c) and pressure physicians can face from patients seeking to access AD (theme 4a) are also well-documented in the international evidence-base.^6,23,37,46–49^ International research also supports the finding that AD providers can have limited legal knowledge of AD legislation (theme 4b).^39^

Though many of the challenges identified in the study reflect international AD experience, on another level, there are aspects of these findings that are specific to Belgium and reflect Belgian regulatory, social, medical and cultural contexts. For example, theme 1 describes challenges associated with the specific framing of the Belgian euthanasia law including its broad eligibility criteria. As another example, challenges associated with patients’ and providers’ lack of knowledge about euthanasia (theme 4) may reflect that the Belgian government has not taken steps to increase community or practitioner awareness about euthanasia.^14^

In addition, some challenges identified in the study have not, to the authors’ knowledge, been identified in the international literature exploring AD. These include the challenge that patients’ and providers’ perceptions of due care in euthanasia assessments can differ (theme 1a) and that providers can find it difficult to apply AD legislation when it is (or has become) incongruous with their usual approaches to clinical decision-making (theme 1b).

Though all challenges identified in the study were experienced by both physician and nurse participants to some extent, some discrete challenges appear to be role-specific. For example, though some physician participants described providing euthanasia as being administratively burdensome, nurse participants did not identify this as a challenge for them. This is likely because physicians are responsible for notifying the FCECE by submitting a registration form after the patient has died. As another example, it was mostly nurse participants who reported challenges associated with health professionals’ lack of knowledge in relation to clinical administration. Nurses are often experts in the clinical administration of euthanasia in Belgium, and no nurse participants reported that they or their nurse colleagues had experienced any technical difficulties with clinical administration. Rather, they reported that physicians can often experience these difficulties, which can present several challenges.

The findings in this study should assist policymakers at the institutional, regional and federal levels to support euthanasia providers and to identify ways by which these challenges can be mitigated. As AD systems move past legalisation, implementation and into ongoing regulation, they evolve.^50–52^ Contemporary evidence of practical challenges is an important source of information for policymakers and can inform reviews of the system, including the need for reform.

The formal, objective evaluation of the law that participants wish for seems needed. Though all general practitioners now receive training on euthanasia as a component of their training, mandatory training for all physicians and nurses should be considered, as both play important roles in the euthanasia process. In addition, efforts should be made to enhance community knowledge about the euthanasia law, including the limitations of the advance request. Consideration should also be given to whether legislative amendment may be needed to clarify provisions of the Act that physicians find hard to apply in practice. Legislators should also reflect on the extent to which reform is needed to better reflect contemporary care provision. Existing research on AD regulation supports that AD regulation needs to be agile and responsive to physicians’ needs.^53^ One area in which this was illustrated in this study was suggestions that the previously dominant *colloque singulier* (decision-making within the confines of the physician-patient relationship) may now be anachronistic, at least in institutional and team-based settings.

Finally, efforts must be made to support isolated practitioners whose experience of these challenges appears to be more acute than those of their colleagues working in the institutional setting. Research investigating the support needs of isolated practitioners should inform these efforts.

### Strengths and limitations

This study had an intentional focus on challenges to physicians’ and nurses’ euthanasia provision. As such, it did not purport to focus on providers’ overall experiences with euthanasia, nor did it seek to highlight facilitators of providing euthanasia.

Though we purposively selected participants, the participants demonstrated little cultural and ethnic diversity. This might indicate that some important perspectives were not included; research suggests that the views of Muslim persons living in Belgium may differ with respect to euthanasia (though this may indicate that there is a paucity of Muslim euthanasia providers who would have been eligible for inclusion in the study).^54^ Despite this, the participants included in the study reflect a diverse cross-section of characteristics relating to profession, gender and experience level. In addition, we included individuals who did not speak English as their first language, implemented measures to ensure effective communication between participants and the research team, and used reflexive reflections on the influence of language on the data to inform theme development.

## Conclusion

This study illuminates the current challenges health professionals experience when providing euthanasia in Belgium. Knowledge of these challenges is important for policymakers at the institutional, regional and national levels. These findings can inform reviews of the system, including the need for regulatory or other reform. Belgian policymakers should consider undertaking a formal evaluation of the law, mandating training for all physicians and making efforts to enhance community knowledge about euthanasia.

## Supplemental Material

sj-docx-1-pcr-10.1177_26323524251318044 – Supplemental material for Key challenges in providing assisted dying in Belgium: a qualitative analysis of health professionals’ experiences Supplemental material, sj-docx-1-pcr-10.1177_26323524251318044 for Key challenges in providing assisted dying in Belgium: a qualitative analysis of health professionals’ experiences by Madeleine Archer, Lindy Willmott, Kenneth Chambaere, Luc Deliens and Ben P. White in Palliative Care and Social Practice

sj-docx-2-pcr-10.1177_26323524251318044 – Supplemental material for Key challenges in providing assisted dying in Belgium: a qualitative analysis of health professionals’ experiences Supplemental material, sj-docx-2-pcr-10.1177_26323524251318044 for Key challenges in providing assisted dying in Belgium: a qualitative analysis of health professionals’ experiences by Madeleine Archer, Lindy Willmott, Kenneth Chambaere, Luc Deliens and Ben P. White in Palliative Care and Social Practice

sj-docx-3-pcr-10.1177_26323524251318044 – Supplemental material for Key challenges in providing assisted dying in Belgium: a qualitative analysis of health professionals’ experiences Supplemental material, sj-docx-3-pcr-10.1177_26323524251318044 for Key challenges in providing assisted dying in Belgium: a qualitative analysis of health professionals’ experiences by Madeleine Archer, Lindy Willmott, Kenneth Chambaere, Luc Deliens and Ben P. White in Palliative Care and Social Practice

## Declarations

**Ethics approval and consent to participate:** The study was reviewed and approved by the Medical Ethics Committee of the Brussels University Hospital with reference BUN 1432022000043; the Queensland University of Technology Human Research Ethics Committee with reference 2000000270. All participants in the study provided written consent to participate (which was confirmed verbally during the interview).

**Consent for publication:** Not applicable.

**Availability of data and materials:** The interview guide used in the study is contained in Supplemental File 2. The data generated and analysed in this study is not publicly available consistent with the study’s ethical approval and the need to ensure participant confidentiality. Queries in relation to the availability of data should be directed to the corresponding author.
