# Supplementary material for: Key challenges in providing assisted dying in Belgium: a qualitative analysis of health professionals’ experiences
Source: Palliat Care Soc Pract. 2025 Feb 6;19:26323524251318044. doi: 10.1177/26323524251318044 (PMC11803728; doi:10.1177/26323524251318044)
Supplement: sj-docx-1-pcr-10.1177_26323524251318044 – Supplemental material for Key challenges in providing assisted dying in Belgium: a qualitative analysis of health professionals’ experiences [file sj-docx-1-pcr-10.1177_26323524251318044.docx]

**Key challenges in providing assisted dying in Belgium: A qualitative analysis of health professionals’ experiences**

**Supplementary Material 1**

This interview guide was used for interviews with both physicians and nurses. Prior to the interview it was adapted to reflect the specific participant’s health profession, work setting, and level of experience providing euthanasia. Not all of these topics presented below were covered in every interview due to the semi-structured nature of the interviews.

**Interview Guide**

Introduction

- Introduce interviewers (including role of Dutch-language facilitating researcher)
- Explanation and confirmation of participant’s eligibility to participate in the study and explanation of the structure and format of the interview
- Thank participant for speaking in English (if participating in English)
- [Microsoft Teams recording, discussion about consent, confidentiality, distress protocols, illegality and the purpose of the interview, obtain demographics information (if not already provided)]

Thank you very much. Do you have any questions about the study before we begin?

1. Overall experience with euthanasia

These initial questions explore your overall experience with euthanasia. As we get started and just to remind you and frame the rest of our conversation, this study is about how the euthanasia regulatory framework is working in practice. This includes the law on Euthanasia, the workings of the Federal Control and Evaluation Commission on Euthanasia, end-of-life consultation centres. We want to know what is working, what is not and how it could be improved.

- To begin, could you please describe the role(s) you have had in the euthanasia cases you have been involved in?
  - Prompts: discussing euthanasia and referring, performing physician, first independent doctor, second independent doctor, other
- What kinds of patients have you assisted in relation to euthanasia?
  - Prompts: terminally ill, non-terminally ill, advance requests, mature minors
- Are you otherwise involved in euthanasia aside from your work as a medical practitioner?
  - E.g. a member of professional organisations, end-of-life consultations, teaching
  - Prompt: could you tell me a little bit about this role and what it involves?

1. Initiating euthanasia

Thank you, these next questions are about how euthanasia starts.

- How does euthanasia for a patient begin?
  - Prompt: Have patients seeking euthanasia raised this topic with you? How do they raise it?
  - Prompt: How did you decide when a patient had made a request for euthanasia?
- [I appreciate that you are now an experienced practitioner, but thinking back to when you were less experienced] once you have/had a patient’s request for euthanasia, how do you know what steps to take next?
- *[If applicable, based on previous discussion regarding participation in euthanasia]* Are there any aspects of euthanasia that you have a conscientious objection to or do not feel comfortable participating in e.g. will assess but not provide etc [especially for participants whose role is confined to the second opinion]. If you feel comfortable to, could you explain why that is?

1. Eligibility assessment process

These next questions are about the process or steps that must be taken for euthanasia. I suppose the basic steps are that first the request is explored, then the first doctor considers whether the person is eligible, they refer to another doctor, there may be a third, then there is the performance – what are your experiences with this process and how it works?

- General prompts:
  - Timeframes
  - Safeguards
  - Reporting and oversight
  - Documentation
  - Finding an advance request for euthanasia
  - Referring
- Specific prompts:
  - First consultation - providing information (therapeutic possibilities, life expectancy, palliative care), finding an independent consultant, ensuring independence, handling positive and negative advices, communicating
  - Specialist consultation for not expected to die within foreseeable future and minors with capacity for discernment - (finding a specialist, how the doctor handles a positive or negative advice re the patient’s eligibility, ascertaining the consultant’s competence, ascertaining whether consultant is a specialist)
  - Minor patients (interview with legal representatives, obtaining consent)
- *[If not already answered]* [Again, thinking back to when you were less experienced] how did/do you know what the process is/was and the next steps to take? Where did/do you get this information from?
- Could you describe any experiences you’ve had with conscientious objection? What I mean by that is other health professionals not wanting to participate in euthanasia due to their personal or religious views.
- Could you describe any experience you have had with non-participation of institutions?
  - Prompts: caused problems in the euthanasia process, by reason of religious affiliation, non-participation for other reasons – how so? To what extent has institutional objection posed a barrier/presented an issue in the provision of euthanasia? How are transfers undertaken?
- I recognise that in some organisations, conditions and steps are added to the law. In your organization/practice are patients required to undergo any steps in addition to the law?
  - Why? What is the function of those additional steps? Who decides what these additional steps are?
    - Prompts: care quality, safety, risk management
  - Why not?

1. Eligibility requirements

Next, I would like to focus on the eligibility requirements or due care criteria for euthanasia, such as unbearable suffering, whether the person’s condition is terminal or non-terminal, decision-making capacity, could you tell me about your experience in applying these requirements? To what extent is this straightforward or complex?

- Reframe: How did you know what the eligibility requirements are, what’s working/not working with applying these requirements, is it difficult, are you supported) – why?
- Prompts: have you had any difficulty making judgements about/assessing eligibility (capacity, voluntariness, medically hopeless condition, constant and unbearable physical or mental suffering, non-alleviability, serious and incurable disorder caused by illness or accident, recurring nature of request, foreseeability v non-foreseeability, irreversibly unconscious (advance directive), capacity for discernment).
- How confident do you feel to make judgements about the patient’s eligibility? How do you overcome any potential uncertainty? How does referral to a colleague work in relation to uncertainty about any of the eligibility criteria?
- Have you experienced any issues in relation to assessing a person’s competence or capacity?

1. Reporting requirements

Now I will ask you about the reporting requirements. By this I mean the requirement to complete the report or declaration and return it to the Federal Commission.

- Could you please talk about any experiences you have in navigating the process by which you submit a declaration and the federal commission reviews it – what works in this process and what doesn’t or what is difficult?
- Prompts (if not covered above)
  - How did you know about the reporting requirements under the euthanasia system?
  - What do you see are the main benefits/issues/challenges going through the euthanasia reporting process?
  - Do you believe the processes are justified? Why? Why not?
  - Do you have any comments on the role of the Federal Control and Evaluation Commission (the Commission) in terms of reporting (or generally)?
  - To what extent do you believe that the Commission’s oversight mechanisms are necessary and effective? How could they be improved?
  - What is your experience using and completing the reporting form? Is the reporting form/reporting system (of half confidential, half disclosed) appropriate? Should it collect more/less information? Do you feel this approach protects physicians? Can you think of any examples of data that is not captured or reported that should be?
  - To what extent do you think the reporting form collects all the information required to assess whether the euthanasia was performed correctly and legally?
  - Are there inefficiencies in the reporting system i.e. registered post not an online portal, for example.
  - [If applicable] How effective is internal (i.e. institutional) oversight and reporting?

1. Prescription of the medication and the performance of euthanasia for eligible patients

The next topic is about actually performing euthanasia.

- *[If applicable]* [When you were less experienced] how did/do you know how to perform euthanasia? Have you yourself experienced or observed any difficulties with the actual performance of euthanasia?
  - Prompt: Did you have any difficulties obtaining the medication?
- How is the decision made about what method of euthanasia to use, whether the doctor will directly administer the medication to the patient or whether the patient might take an oral form of the medication?
- What factors and rules (if any) influenced your decision for self-administration (physician assisted suicide) or practitioner administration (voluntary euthanasia)? In your experience, do patients indicate a preference one way or the other? Do you think that both options should be available?
- What aspects of the process for performing euthanasia work well and what needs improvement?
  - Prompts: medication supply, training, self-administration v practitioner administration, adverse events
- Have you experienced any issues with conscientious objection from other healthcare professionals (e.g. pharmacist for dispensing the medication) or institutional objections? (including at other stages of the process?)

1. Sources of information about steps in the process

This next set of questions explores the sources of information you have used in providing euthanasia, like documents, policies, and training – so first:

- *[If not already covered*] How did you find out about the steps in the euthanasia assessment process? Where did you get this information from?
  - Prompt: How did you know what eligibility criteria to use to assess eligibility and where did that come from? How did you know what was the first step, what was the second, when a third consultation was needed, for example?
- *[If not already covered*] How did you find out about the reporting requirements?
- What documents guide you in making decisions about euthanasia?
  - Prompts: law, policy, guidelines, ethical codes, training, professional norms, FCECE brochure, documents produced by your palliative care network, anything else?
  - How do they guide you to make decisions about euthanasia?
  - Would you be willing to provide access to such sources (if appropriate)?
  - Does your organisation/network provide any training in relation to euthanasia?
  - If you belong to a professional organisation, do they provide any guidance on euthanasia that is useful? E.g. the Order of Physicians, specialist organisation, Palliative Care Flanders.
- What bodies guide you in making decisions about euthanasia?
  - Prompts: colleagues, ethics committees, hospitals, LEIF, health funds, medical college?
  - How do they guide you?
  - Is their guidance useful for you when making decisions about euthanasia?
  - Would you be willing to provide access to such guidance (if appropriate)?
  - Who is involved in the euthanasia decision-making process? Any involvement from the hospital ethics committee?
- How influential are these sources, people and institutions in how you make decisions about euthanasia?
  - Prompt: which is most important?
- How useful are these sources, people, or institutions when making decisions about euthanasia?
- How do you manage any competing or conflicting guidance in these sources, people, or institutions?
- Where there is disagreement or uncertainty about the processes to be followed or whether someone is eligible to access euthanasia, what steps would you take to resolve that disagreement or uncertainty?
- What resources do you use when supporting other doctors with euthanasia, for example when they need further support or information on an aspect of practice?
- When the law is ambiguous or vague, how do you decide how you act? How do you fill those gaps?
  - Prompt: colleagues, LEIF, consultation centre?
  - Is it more your ethics? Clinical discretion? Peer consultation?

1. Patient perspectives

Now let’s touch briefly on the experiences patients have with euthanasia.

- In your practice are you aware of any particular difficulties that your patients or their families have faced in the euthanasia process? What are they?
- Prompts: difficulty in finding a physician, waiting periods, lack of support, additional/different requirements, institutional objection, conscientious objection.

1. Interactions between euthanasia and palliative care

Let’s briefly consider how euthanasia and palliative care intersect.

- Could you explain the relationship between euthanasia and palliative care in Belgium?
- To what extent are euthanasia and palliative care integrated? Well? With difficulty?

1. Regulatory system’s ability to meet policy goals in practice

Now to ‘zoom out’ and take a broad view of the euthanasia system.

- What works well with the current system of euthanasia? What is the best aspect of the euthanasia system/structure?
- What aspect of the euthanasia process/structure that needs the most improvement? What are existing challenges in the way that euthanasia is regulated/practised in Belgium?
- Many would agree that in Belgium doctors are given a fair bit of discretion in their euthanasia practice which might lead to different professionals doing things differently, still within the bounds of the law. To what extent do you think this is a good thing or a bad thing?
- What are your views on euthanasia for persons who are tired of life – does regulation need to be changed?
- Similarly, for people with advanced dementia, does regulation need to be changed?
- How could the current regulation of euthanasia be improved? What are barriers?
  - What matters are currently regulated that shouldn’t be?
  - What matters aren’t currently regulated that should be?
- We have spoken a lot about the processes of euthanasia but what about the rules around who can have access to it in the first place (explain eligibility criteria). Are these the right groups who should have access under the law? Or should it be broadened or narrowed?
- Are the legal due care criteria appropriate?

Prompts: too specific, too vague, too narrow, too limiting, not effective.

Have you experienced any difficulties in translating the law or policy into clinical practice? Has anything assisted with this translation?

- In Australia, there are limitations on the extent to which doctors and health professionals can raise euthanasia with their patients – what do you think of this?
- How has the 2020 amendment to the law impacted your decision-making about euthanasia?
- Reframe: are you aware of any changes to the law in recent times and how have these impacted your euthanasia practice at all?
  - Prompts: individual referral obligation, institutional objection, non-expiry of advance directives.
- How have court decisions impacted your decision-making about euthanasia?
  - Reframing: Are you aware of any euthanasia litigation and how has this affected your practice?
  - Prompt: constitutional cases or criminal cases?
  - How do you think that trial has impacted the work of other euthanasia providers? Has it impacted the broader euthanasia landscape at all?
- How long does getting through the euthanasia process take from first request through to accessing the medication? Is this appropriate? Euthanasia involves a system that has safeguards to ensure only those who are eligible have access to euthanasia while facilitating reasonable access for those who qualify. How do you think the current euthanasia regulation strikes this balance?
- Some research suggests that the law is not always complied with in relation to euthanasia. Do you have any thoughts on why this might be the case? Without giving any specific examples?
  - Prompts: law is vague, professional judgement, paternalistic attitudes, anti-social control of euthanasia, rejection of regulation, depends on case-by-case assessment, law is too burdensome, euthanasia is a private matter.
- Some research suggests that not all cases of euthanasia are reported. Do you have any thoughts on why this might be the case, without giving any specific examples?
  - Prompts: the reporting process is difficult/burdensome, lack of compliance with the process in practice so won’t report that, technology failures, euthanasia is a private matter.
- Autonomy and safety are two important principles in assisted dying regulatory frameworks. What do you think are the principles that should underpin the Belgian euthanasia system? To what extent are these achieved in practice?
  - Reframe: In the Australian systems, patient safety appears to be the key concern so there are very strict processes and eligibility criteria. In Belgium, there seems to be a different balance – perhaps patient autonomy is more central? Do you have any thoughts on this?

1. Macro perspectives

To finish, I want to ask you a question that is a little more abstract or theoretical. The Government wants doctors to follow the rules for providing euthanasia and so it has set up various rules i.e. the euthanasia law, there are policies and guidelines, there is training and other support and systems to try and make sure things happen as planned. If you are trying to design a system that doctors will listen to and follow, what is the most effective way to guide doctors’ behaviour when providing euthanasia? What sorts of ‘rules’ are doctors most likely to listen to, what will they follow in providing euthanasia?

- Prompt: law, policy, guidelines, ethical codes, training, professional norms, anything else?
- Which of these are most likely to influence your decisions about providing euthanasia?
- What makes you follow rules or not follow rules?
- How can we best reach health professionals?
- Putting aside the current euthanasia system, if you could tell those designing a new euthanasia system who wanted it to be the best system possible some advice, what advice would you give?
- Reframing: Do you have any advice from the Belgian framework about what new countries to euthanasia should and shouldn’t do?
  - What principles or values do you think should underpin that system? For example, beneficence, autonomy, safety?
  - What features do you think the system should have?

1. Close

We are giving participants the option to review their interview transcript if they would like to do so. We will send the transcript to you once it is completed and you can choose to check it for accuracy or to add any comments if you would like, but you don’t have to. If we don’t hear from you in around 2 weeks we will presume you are happy with the transcript.

Would you like to receive information about the result of the study? If so, what is your preferred email or postal address?

If any aspect of this interview has caused you distress, the information and consent form includes some support options you are available to contact. Has the interview caused you to experience distress?

Do you know anyone else who might be eligible and willing to participate in this study?

Do you have any questions for me about the study or anything else? You’ll be hearing from me soon.

Thank you very much for taking part in this interview.
