# Supplementary material for: Key challenges in providing assisted dying in Belgium: a qualitative analysis of health professionals’ experiences
Source: Palliat Care Soc Pract. 2025 Feb 6;19:26323524251318044. doi: 10.1177/26323524251318044 (PMC11803728; doi:10.1177/26323524251318044)
Supplement: sj-docx-2-pcr-10.1177_26323524251318044 – Supplemental material for Key challenges in providing assisted dying in Belgium: a qualitative analysis of health professionals’ experiences [file sj-docx-2-pcr-10.1177_26323524251318044.docx]

**Key challenges in providing assisted dying in Belgium: A qualitative analysis of health professionals’ experiences**

**Supplementary Material 2**

**Consolidated criteria for reporting qualitative studies (COREQ): 32-item checklist**

| **COREQ item** | **Discussion of this item** | **Section of the article which presents this information** |
| --- | --- | --- |
| Domain 1: Research team and reflexivity | | |
| 1. Interviewer/facilitator | MA led the interviews in which the participant was comfortable speaking English, assisted in 4 interviews by LW.  MA led the interviews in which the participant was comfortable speaking in English with a Dutch-speaking member of the research team present to facilitate, which was KC in 3 interviews, and LD in 1 interview.  KC led the interviews in which the participant was comfortable speaking in Dutch. | Methodology |
| 1. Credentials | MA: BSc-LLB (First Class Hons), GradDipLegPrac | N/A |
| 1. Occupation | MA is a PhD candidate in the Australian Centre for Health Law Research at the Queensland University of Technology. | N/A |
| 1. Gender | Female | N/A |
| 1. Experience and training | MA has a number of years of experience conducting research on assisted dying in Australia and in Belgium. Prior to the commencement of the study, MA received training in conducting qualitative interviews. The other members of the research team have extensive experience in conducting qualitative interviews and were present in several of the interviews to facilitate. | N/A |
| 1. Relationship established | As one method of participant recruitment was recruitment through the professional networks of the research team, some of the participants were known to some members of the research team prior to the interviews. MA did not have any prior relationship with the study participants prior to the interviews. KC did not have any prior relationship with the study participants who participated in an interview in Dutch. | N/A |
| 1. Participant knowledge of the interviewer | The aims and purposes of the research conducted was presented to participants in the informed consent form which they were required to read before signing. At the beginning of each interview, the aims and purposes of the study were again explained to participants, and they were given the opportunity to ask any questions that they had about the study before, during, and after the interview. | N/A |
| 1. Interviewer characteristics | Participants were informed prior to the interview that this research is part of a broader study of assisted dying legislation being conducted in Australia. Participants were informed that this specific study was being conducted for MA’s PhD project which focuses on Belgian euthanasia regulation. | N/A |
| Domain 2: Study design | | |
| 1. Methodological orientation and theory | This study involved semi-structured qualitative interviews analysed using a reflexive approach to thematic analysis. | Methodology |
| 1. Sampling | Participants were recruited directly through the research team’s professional networks, advertisements disseminated by relevant organisations, and through snowball approaches to recruitment. | Methodology |
| 1. Method of approach | Participants were contacted via email, or they made initial contact with a member of the research team via email. | N/A |
| 1. Sample size | In total, 20 interviews were conducted. | Methodology |
| 1. Non-participation | No participants withdrew from the study subsequent to participation. | N/A |
| 1. Setting of data collection | Participants were able to choose the location from which they attended the interview, which for most participants was from their workplace or from their home. | N/A |
| 1. Presentence of non-participants | There were no individuals present in any of the interviews in addition to the participant and a member(s) of the research team. | N/A |
| 1. Description of sample | Participant demographic information is presented in the results section of the article, which reports on a number of participant characteristics. | Results |
| 1. Interview guide | MA used an interview guide which was adapted slightly for each interview to reflect the participant’s experience providing euthanasia and the work setting. The broad discussion areas contained in the interview guide were provided to participants prior to the interview if they requested it. | Methodology; Supplementary Material 1 |
| 1. Repeat interviews | There were no repeat interviews conducted for this specific study. | N/A |
| 1. Audio/visual recording | Microsoft Teams videoconferencing was used in the interviews. The interviews were recorded to enable subsequent transcription. | Methodology |
| 1. Field notes | Field notes were made during and after the interview. Reflexive notes were made after the interview. Both the field and reflexive notes were used to inform subsequent data analysis. | Methodology; Results |
| 1. Duration | The median length of interviews was 94 minutes (with a range of 65-111 minutes). | Results |
| 1. Data saturation | No further interviews were conducted once MA and KC determined that ongoing analysis demonstrated sufficient ‘information power’ to answer the study’s research questions. | Methodology |
| 1. Transcripts returned | Participants were sent their transcripts once it had been prepared and they were informed that they could add any information to it, alter any information, or remove any information from it. | Methodology |
| Domain 3: Analysis and findings | | |
| 1. Number of data coders | MA coded the data. The research team discussed the coding at several stages, which resulted in some themes being merged, adapted, and settled. | Methodology |
| 1. Description of the coding tree | A description of the coding tree has not been included. | N/A |
| 1. Derivation of themes | Themes were derived from the data; the code and theme development processes were inductive rather than deductive, as described in the methodology section of the article. | Methodology |
| 1. Software | NVivo (release 1.6.1) was used to facilitate data analysis. | Methodology |
| 1. Participant checking | Participants did not give feedback on the findings. | N/A |
| 1. Quotations presented | Participant quotations are presented to illustrate the themes. Each participant quotation indicates that participant’s unique participant identification number. | Results |
| 1. Data and findings consistent | Yes. |  |
| 1. Clarity of major themes | Major themes are clearly presented in the results section. | Results |
| 1. Clarity of minor themes | A description of diverse cases and minor themes feature in the results section. | Results |
