# Supplementary material for: Key challenges in providing assisted dying in Belgium: a qualitative analysis of health professionals’ experiences
Source: Palliat Care Soc Pract. 2025 Feb 6;19:26323524251318044. doi: 10.1177/26323524251318044 (PMC11803728; doi:10.1177/26323524251318044)
Supplement: sj-docx-3-pcr-10.1177_26323524251318044 – Supplemental material for Key challenges in providing assisted dying in Belgium: a qualitative analysis of health professionals’ experiences [file sj-docx-3-pcr-10.1177_26323524251318044.docx]

**Key challenges in providing assisted dying in Belgium: A qualitative analysis of health professionals’ experiences**

**Supplementary Material 3**

**Reflexive Thematic Analysis Reporting Guidelines (RTARG) – Virginia Braun & Victoria Clarke**^[[1]](#footnote-1)^

This supplementary material is included to provide more information on the consideration given to the Reflexive Thematic Analysis Reporting Guidelines (‘RTARG’) in the conduct and reporting of this study. Braun and Clarke’s advice for each section of a research paper which uses reflexive thematic analysis (‘RTA’) is presented below, with a corresponding discussion of that advice and how it was reflected in the conduct and reporting of the study (where feasible and appropriate).

| **RTARG advice** | **Discussion of this advice and how it is reflected in this article, where feasible and appropriate** |
| --- | --- |
| **The Introduction** | |
| ***Background and rationale*** | |
| Provide a robust context and rationale for the proposed research in the Introduction. | The introduction section of the article discusses existing research, relevant regulatory theory and provides context in relation to this research on the key challenges euthanasia providers in Belgium experience in their practice. This section of the article identifies how the new knowledge generated in this article adds to and enriches existing knowledge that exists by seeking to investigate a broader spectrum of challenges than previously explored. |
| Clearly articulate a research question – one that is methodologically coherent | The introduction section of the article presents a clear statement of the article’s methodologically coherent research question. It is: ‘what are the key challenges that health professionals experience when providing euthanasia in Belgium?’ This research question is appropriately answered through the semi-structured interview study described in the methodology section of the article. |
| ***“Owning your own perspectives”*** | |
| Include information on guiding theoretical assumptions and other (e.g. explanatory) theory informing use of TA) | We adopt a critical realist ontological and epistemological positioning in this research. This is stated in the Methodology section of the article. |
| Report in a way that is consistent with stated theoretical assumptions throughout | We have made efforts to report this research in a way that is consistent with the critical realist positioning of the research. |
| Evidence methodological coherence/integrity in both the research and the report | We have made efforts to evidence methodological coherence in the research conducted for and reported in this article. |
| Show evidence of reflexive practice | The Methodology section of the article acknowledges researcher reflexivity and notes the use of a reflexive journal in the coding and theme development processes. Further information about researcher reflexivity is provided below. |
| Write in a methodologically coherent style | We have made efforts to write the article in a way that reflects the researchers as active contributors to and influences upon the research. |
| **The Methodology** | |
| ***Participants/data items*** | |
| Describe selection of participants/data items | We describe the selection of participants in the Methodology section. |
| Describe number of participants/data items; provide a rationale or explanation around dataset or participant group size/composition | The number of participants and a rationale around the participant group size and composition is provided in the Methodology section. This study used the non-positivist qualitative concept of ‘information power’ to determine the point at which interviews ceased. |
| Discuss characteristics of participants/data items | The characteristics of the selection of participants included in the study are presented in the Results section of the article. This information is provided in aggregate form in Table 2. Though we acknowledge that aggregating this information limits the extent to which each participant’s characteristics can be understood in the context of the other participants, we consider that the information provided effectively situates each participant in the whole group of participants. In addition, this reflects that the most important consideration with this data was the need to ensure participant anonymity. |
| Detail ethical approval and ethical code/principles followed, participant informed consent, etc | Given length constraints, information on the ethical approval that the study received, and the provision of informed consent is provided in the sections following the article. |
| ***Dataset generation*** | |
| Provide some rationale for method(s) for data generation/data item sources chosen | Semi-structured interviews are a useful method for data generation in this study. Semi-structured interviews allow the researchers to follow a relatively structured approach to the interview (and the use of an interview guide to guide the discussion) while permitting divergences from that structure when the participant shares insights or perspectives that are unique or warrant a deeper exploration. |
| Describe development and/or characteristics of data generation tools | We developed and used an interview guide in this study which guided data generation. This interview guide is described in the Methodology section of the article and is provided in Supplementary Material 1. |
| Include details such as modality and/or setting of data generation, time frame, and other pertinent procedural information | We undertook semi-structured interviews using Microsoft Teams videoconferencing software at the time and location of the participant’s choosing. To reflect the time difference between Australia and Belgium, participants were given the option of participating in the morning or in the evening at a specific time they selected, and which suited them. Interviews were undertaken from September 2022 to March 2024. This is described in the Methodology section of the article. |
| Describe who conducted any interactive data generation (which author or research role), and how | The roles that each researcher had in the data generation process is described in the Methodology section. |
| Describe the size/scope of the dataset and dataset items | The range and median length of the interviews are described in the Results section. |
| Describe, and if relevant explain, any preparation of data for analysis | The use of transcription and translation of the interviews undertaken in Dutch is described in the Methodology section. The use and function of obtaining further reflections from participants on their transcript is also described in that section. |
| ***Data analysis*** | |
| Provide some rationale for the use of RTA, and were relevant, for combining RTA with other approaches and procedures | The rationale for using RTA is described in the Methodology section. |
| Describe specific orientation to RTA | The inductive orientation to reflexive thematic analysis is described in the Methodology section. |
| Discuss how the researcher(s) engaged with the analytic process | MA analysed the data, with guidance from the other authors. MA followed the six phases of Braun and Clarke’s reflexive thematic analysis, consisting of: data familiarisation, coding, generating initial themes, developing and reviewing themes, refining, defining and naming themes, and writing up.^[[2]](#footnote-2)^ Themes were settled following discussions within the research team. |
| Where more than one person is involved, describe who analysed the data (author or research role) | MA primarily analysed the data. Several discussions took place within the research team to discuss the framing, content, and relationship between themes. Inter-coder agreement measures and consensus coding approaches were not used, being inconsistent with the theoretical assumptions underlying the research. |
| Use language to describe the process and products of RTA that is coherent with the values and assumptions of RTA | The language used in the Methodology section is coherent with the values and assumptions of RTA. For example, the Methodology section of the article describes how themes were ‘generated’ or ‘constructed’ by the researchers through the analytical process rather than ‘discovered’. Though Braun and Clarke prefer the term ‘Analysis’ to ‘Results’ (to describe the section in which the themes as analytic outputs are presented), the term ‘Results’ is used in this study as this term is likely to be the most familiar for readers. In addition, we considered that naming the section ‘Analysis’ may have lead readers to view that section as further elaboration of the methodology instead of a discussion of analytic outputs. |
| **The Analysis** | |
| ***Reporting the data analysis*** | |
| Provide an overview of themes or thematic structure | An overview of themes and the thematic structure (including the relationship between themes and sub-themes) is included in the Results section of the article. This overview is consistent with the narrative about the themes that follows it. |
| Ensure theme conceptualisation is appropriate to RTA, and any divergences are justified and explained | Themes generated in this study reflect patterns of shared meaning, rather than topic summaries. Some of the themes generated in the analysis are sematic and some are latent. |
| Name themes appropriately | The theme names reflect the essence of each theme. |
| Report themes in sufficient detail and depth | The themes are reported in considerable detail and depth; data extracts (illustrative participant quotations) are included, and the reporting of themes references both participant data and the researchers’ narrative in relation to that data. |
| Use subtheme judiciously | The themes are multifaceted and complex. Some sub-themes were generated during data analysis. These ‘levels’ of themes were developed judiciously and reflect the data. |
| Ensure analytic narrative explains the meaning and significance of the data | The analytic narrative explains participant data (encompassing an integration of participants’ data and the researchers’ analytical narrative regarding that data). Descriptions are provided for how illustrative participant quotations (data extracts) provide evidence for the statements made in the reporting of themes. |
| Provide an appropriate balance of analytic narrative and data extracts – both data extracts and analytic narrative matter | The reporting of the themes integrates participants’ data and the researchers’ analytical narrative relating to that data. Participant quotations are provided to illustrate the themes. |
| Demonstrate coherence between analytic narrative and illustrative/evidentiary abstracts | The analytic narrative and data extracts (participant quotations) are coherent. |
| Integrate existing research and theory into the analytic narrative | Existing research and theory are not integrated in the analytic narrative (presented in the Results section of the article). The Discussion section is the section in which the analysis in the study is integrated with existing research and theory. We recognise that Braun and Clarke recommend that this integration occurs. However, this decision was taken to reflect and reinforce the inductive (not deductive) orientation to theme development used in the study and to avoid confusion that a deductive approach to analysis was taken. |
| **The Final Section – A General Discussion or “Conclusions”** | |
| ***Quality, evaluation and conclusions*** | |
| Draw analytic conclusions across themes | The Discussion section of the article discusses the overall implications of the research and describes the narrative that integrates each of the themes. Suggestions for policy reform are included in this section. |
| Discuss implications or directions for future research | Suggestions for future research are included in the Discussion section. Implications of the research for policy are also included in the Discussion section. |
| Use and report quality practices coherent with RTA | Due to lengths constraints, the article does not describe the use of quality practices coherent with a reflexive approach to thematic analysis. However, these quality practices were used in the research design. For example, the Limitations section of the Discussion acknowledges that some participants were not interviewed in their mother tongue, though measures were implemented to ensure that their insights were understood and accurately reported by the researchers. MA maintained a reflexive journal throughout the coding and theme development processes that included reflections on the implications of this linguistic difference on the data. In addition, MA also made reflections in the journal regarding the external positioning of some members of the research team with respect to the participants’ cultural, linguistic, geographical, and other settings (as well as the positioning of other members of the research team being embedded in participants’ local culture). These reflections in the reflexive journal were used to inform coding and theme development. |
| Evaluate the research from a Big Q standpoint | The Discussion section of the article evaluates this study from a Big Q standpoint. |
| Include reflections on research process and practices, including researcher reflexivity | Given length constraints, it was not possible to include reflections about researcher reflexivity in the Discussion section of the article. Please see the above response with respect to researcher reflexivity. |

1. Virginia Braun and Victoria Clarke, ‘Supporting Best Practice in Reflexive Thematic Analysis Reporting in Palliative Medicine: A Review of Published Research and Introduction to the Reflexive Thematic Analysis Reporting Guidelines (RTARG)’ (2024) 38 Palliative Medicine 608. [↑](#footnote-ref-1)
2. Virginia Braun and Victoria Clarke, *Thematic Analysis: A Practical Guide* (SAGE Publications 2022). [↑](#footnote-ref-2)
